# Inferring risks of coronavirus transmission from community household data

**DOI:** 10.1177/09622802211055853

**Published:** 2022-09-10

**Authors:** Thomas House, Heather Riley, Lorenzo Pellis, Koen B Pouwels, Sebastian Bacon, Arturas Eidukas, Kaveh Jahanshahi, Rosalind M Eggo, A. Sarah Walker

**Affiliations:** 1 Department of Mathematics, 5292 University of Manchester, Manchester UK; 2 IBM Research, Hartree Centre, Daresbury UK; 3 The Alan Turing Institute for Data Science and Artificial Intelligence, London UK; 4 105596 Nuffield Department of Medicine, University of Oxford, Oxford UK; 5 The National Institute for Health Research Health Protection Research Unit in Healthcare Associated Infections and Antimicrobial Resistance at the University of Oxford, Oxford UK; 6 Health Economics Research Centre, Nuffield Department of Population Health, University of Oxford, , Oxford UK; 7 The DataLab, 12205 Nuffield Department of Primary Care Health Sciences, University of Oxford, Oxford UK; 8 Data Science Campus, Office for National Statistics (ONS); 9 Centre for Mathematical Modelling of Infectious Diseases, 4906 London School of Hygiene and Tropical Medicine, London UK; 10 The National Institute for Health Research Oxford Biomedical Research Centre, University of Oxford, Oxford UK; 11 MRC Clinical Trials Unit at UCL, UCL, London UK

**Keywords:** Epidemic, COVID-19, model, infection, risk factors

## Abstract

The response of many governments to the COVID-19 pandemic has involved measures to control within- and between-household transmission, providing motivation to improve understanding of the absolute and relative risks in these contexts. Here, we perform exploratory, residual-based, and transmission-dynamic household analysis of the Office for National Statistics COVID-19 Infection Survey data from 26 April 2020 to 15 July 2021 in England. This provides evidence for: (i) temporally varying rates of introduction of infection into households broadly following the trajectory of the overall epidemic and vaccination programme; (ii) susceptible-Infectious transmission probabilities of within-household transmission in the 15–35% range; (iii) the emergence of the Alpha and Delta variants, with the former being around 50% more infectious than wildtype and 35% less infectious than Delta within households; (iv) significantly (in the range of 25–300%) more risk of bringing infection into the household for workers in patient-facing roles pre-vaccine; (v) increased risk for secondary school-age children of bringing the infection into the household when schools are open; (vi) increased risk for primary school-age children of bringing the infection into the household when schools were open since the emergence of new variants.

## Introduction

### Analysis of household infection data

Households have often played an important role in infectious disease epidemiology, with policies in place and under consideration in the UK to reduce both within- and between-household transmission.^1^ This is because the close, repeated nature of contact within the household means that within-household transmission of infectious disease is common. Also, most of the population lives in relatively small, stable households.^2^ From the point of view of scientific research, the household is a natural unit for epidemiological data collection and households are small enough to allow for explicit solution of relatively complex transmission models. Some of the earliest work in this field was carried out by Reed and Frost, whose model was first described in the literature by Abbey^3^ in a paper that analysed transmission in boarding schools and other closed populations. Frost’s 1928 lecture was later published posthumously,^4^ with a re-analysis of his original household dataset from the 1918 influenza pandemic carried out using modern computational and modelling approaches by Fraser et al.^5^

Subsequent important contributions were made in empirical studies of transmission in households, for example the highly influential study of childhood diseases by Hope Simpson,^6^ and epidemic theory based on the analyses of discrete- and continuous-time Markovian epidemics presented by Bailey.^7^ A key development was the solution by Ball^8^ of the final size distribution of a random epidemic in a household without requiring Markovian recovery from infection, which then enabled statistical analyses of household infection data such as that by Addy et al.^9^ Still further progress is possible due to the use of modern computational methods, particularly Monte Carlo approaches, to augment datasets^10–12^ or to avoid likelihood calculations.^13^

Continued methodological developments and data availability have enabled increasingly sophisticated inferences to be drawn from household studies of respiratory pathogens, dealing with for example interactions between adults and children,^14^ case ascertainment,^15^ interactions between strains,^16^ and details of family structure.^17^ During the current pandemic, there have been numerous household studies,^18^ with three recently published studies being notable for combining fitting of a transmission model with significant differentiation of risks being those of Dattner et al.,^19^ Li et al.^20^ and Reukers et al.^21^

### Context for this study

The severe acute respiratory syndrome coronavirus 2 (SARS-CoV-2) emerged in the human population in late 2019 and the WHO declared a pandemic in March 2020.^22^ Early in the pandemic, it became clear that risks of transmission, mortality and morbidity from the associated coronavirus disease (COVID-19) were highly heterogeneous with age,^23^ and also that work in patient-facing roles was associated with increased risk of positivity in the community^24^ as would be expected given the risks of healthcare-associated transmission.^25^

During the period of the study, there have been two major ‘sweeps’ in the UK, during which a SARS-CoV-2 variant of concern (VOC) emerged and became dominant.

The first of these was PANGO lineage B.1.1.7,^26^ or ‘Alpha’ under WHO nomenclature.^27^ The first samples of this variant were found in September 2020,^26^ and it was designated a VOC on 18 December 2020.^28^ There is evidence for both increased transmissibility of this variant, and increased mortality amongst infected cases,^29–31^ although conditional on hospitalisation outcomes may not be worse.^32^ The second VOC to emerge was PANGO lineage B.1.617.2,^33^ or ‘Delta’ under WHO nomenclature,^27^ which was designated a VOC on 6 May 2021 and is now the dominant variant in the UK.^34^

Both of these variants were relatively easy to track through the S gene target in commonly used polymerase chain reaction (PCR) tests, with more details on this approach provided in section ‘Description of data’sec below.

Throughout 2021, the UK rolled out a comprehensive vaccination programme with priority given to healthcare workers, the clinically vulnerable, and then with prioritisation by age, from oldest to youngest.^35,36^ We will not include vaccination here at the individual level, but rather note its overall effect on infection and transmission at different times.

Here, we apply a combination of methods, including a regression that explicitly accounts for transmission, to the Office for National Statistics (ONS) COVID-19 Infection Survey (CIS) data from 26 April 2020 to 15 July 2021.^24^ We particularly consider the absolute magnitude of transmission within and between households, as well as the associations between these and household size, age, infection with VOCs (inferred via S gene target) and work in patient-facing roles.

## Methods

### Description of data

ONS CIS^1^ has a design based on variable levels of recruitment by region and time as required by policy, but otherwise uniformly random selection of households from address lists and previous ONS studies on an ongoing basis. If verbal agreement to participate is obtained, a study worker visits each household to take written informed consent, which is obtained from parents/carers for those aged 2–15 years. Participants aged 10–15 years provide written assent and those under 2 years old are not eligible.

**Figure 1.**
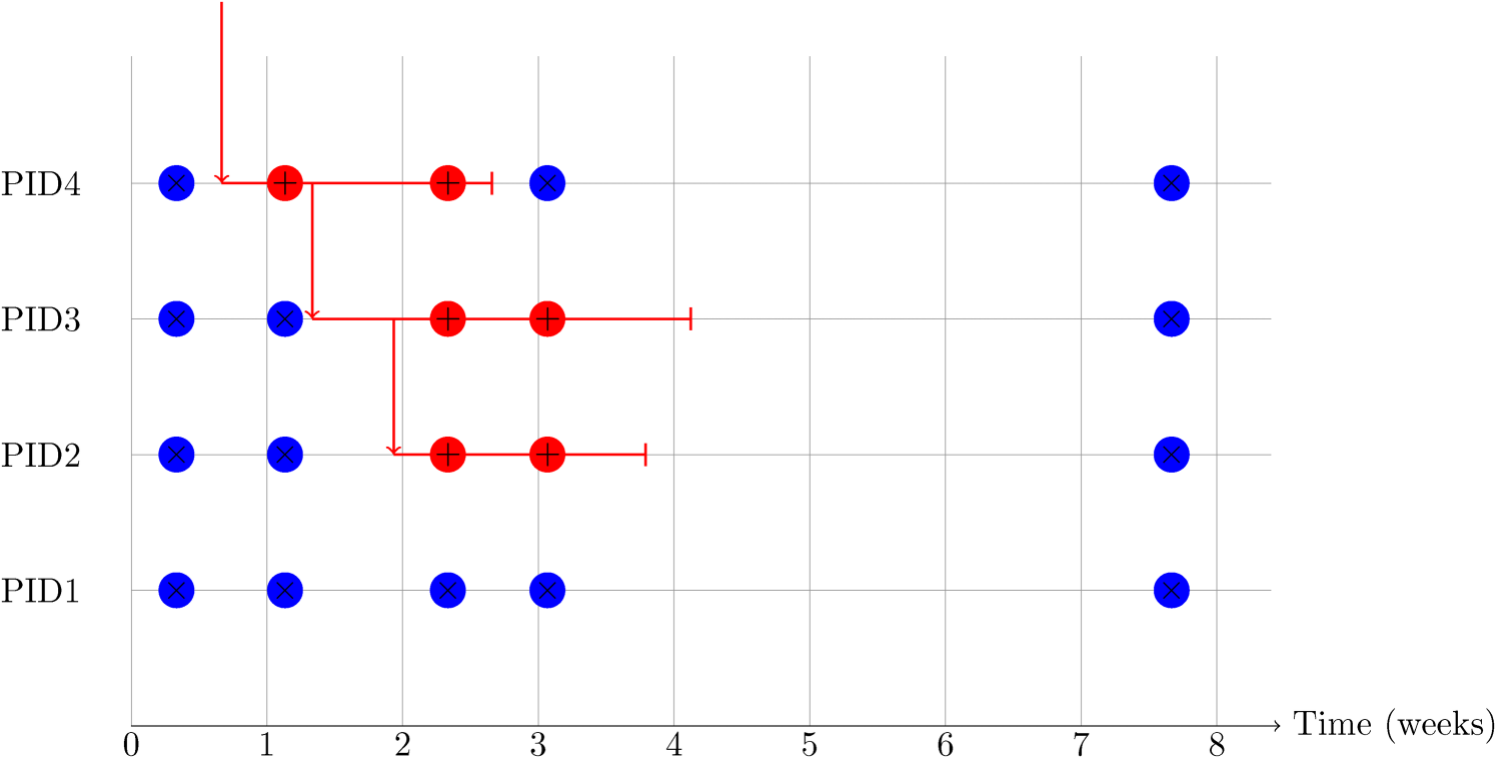
Schematic diagram of a hypothetical but realistic data pattern for a four-person household in the first 2 months after recruitment. Each negative test is shown as a blue circle containing × and each positive test is shown as a red circle containing + . One potential route for infection coming into and transmitting within the household is shown as through a series of red arrows. This is not directly observed in the study design, and in fact other transmission trees (for example, one in which PID2 is infected before PID3) are consistent with the data that would be obtained from this household.

Participants are asked questions on issues including work and age^2^ as well as being tested for SARS-CoV-2 infection via reverse transcription PCR (RT-PCR). To reduce transmission risks, participants aged 12 years and over self-collect nose and throat swabs following study worker instructions, and parents/carers take swabs from children aged under 12 years. At the first visit, participants are asked for optional consent for follow-up visits every week for the next month, then monthly for 12 months from enrolment. The first few weeks of a hypothetical household participating in this study are shown schematically in Figure 1.

Swabs were analysed at the UK’s national Lighthouse Laboratories at Milton Keynes and Glasgow using identical methodology. RT-PCR for three SARS-CoV-2 genes (N protein, S protein and ORF1ab) used the Thermo Fisher TaqPath RT-PCR COVID-19 Kit, and analysed using UgenTec FastFinder 3.300.5, with an assay-specific algorithm and decision mechanism that allows conversion of amplification assay raw data from the ABI 7500 Fast into test results with minimal manual intervention. Samples are called positive if at least a single N-gene and/or ORF1ab are detected. Although S gene cycle threshold (Ct) values are determined, S gene detection alone is not considered sufficient to call a sample positive.

This analysis includes all SARS-CoV-2 RT-PCR tests of nose and throat swabs from 26 April 2020 to 15 July 2021 for English households in the ONS CIS. We restrict our analysis to households of size 6 and under, partly for computational reasons that we will discuss below, and partly because this captures the overwhelming majority of households, with larger households being atypical in various ways.^2^ Over 94% of households have all members participating, and for the remainder we treat the household as composed of participants only. In contrast to other studies, the households we select constitute an approximately representative sample from the population when stratified by date and region. The restriction to England was chosen because we split the data into four time periods, corresponding to changing situations about policies that are devolved (i.e. policies are different in Scotland, Wales and Northern Ireland). These time periods split the data into the following tranches, with associated time periods and notable events (described broadly).

- Tranche 1: 26 April 2020 to 31 August 2020; low prevalence; schools closed; Alpha and Delta variants not emerged yet; no vaccine available.
- Tranche 2: 1 September 2020 to 14 November 2020; high prevalence; schools open; negligible Alpha variant; Delta variant not emerged yet; no vaccine available.
- Tranche 3: 15 November 2020 to 31 December 2020; high prevalence; schools open; Alpha variant becomes dominant; Delta variant not emerged yet; negligible vaccine coverage.
- Tranche 4: 1 January 2021 to 14 February 2021; high prevalence; schools closed (except for pre-school); Alpha variant dominant; Delta variant not emerged yet; over 10 million first vaccine doses by end of time period.
- Tranche 5: 15 February 2021 to 29 April 2021; low prevalence; schools open; Delta variant negligible; over 35 million first and 15 million second vaccine doses by end of time period.
- Tranche 6: 30 April 2021 to 15 July 2021; high prevalence; schools open; Delta variant becomes dominant; over 45 million first and 35 million second doses distributed by end of time period.

These properties are summarised again in Table 1. The properties of the data allocated to these tranches are shown in Table 2. Note that, while we do not include new primary infections in households after 15 July 2021, but do include later secondary infections in households where the primary infection happened before 15 July 2021. This is done to reduce problems with censoring.

**Table 1.** Summary of properties of the time periods (tranches) that the data are split into for analysis.

Tranche	Start date	End date	Prevalence	Schools	Alpha variant	Delta variant	Vaccination
1	26-Apr-20	31-Aug-20	Low	Closed	Not emerged	Not emerged	None
2	1-Sep-20	14-Nov-20	High	Open	Negligible	Not emerged	None
3	15-Nov-20	31-Dec-20	High	Open	Becomes dominant	Not emerged	Negligible
4	1-Jan-21	14-Feb-21	High	Mainly closed	Dominant	Not emerged	>10 M 1st, negligible 2nd
5	15-Feb-21	29-Apr-21	Low	Open	Dominant	Negligible	>35 M 1st, >15 M 2nd
6	30-Apr-21	15-Jul-21	High	Open	Declining	Becomes dominant	>45 M 1st, >35 M 2nd

**Table 2.** Features of the dataset and different tranches.

	Tranche 1	Tranche 2	Tranche 3	Tranche 4	Tranche 5	Tranche 6	Overall
Number of participants	89,624	293,570	315,187	329,532	343,821	351,879	408,278
Number of households	43,300	144,904	157,432	165,238	171,809	178,955	200,876
Number of positive individuals	242	5625	6078	6925	1440	1890	23,392
Households with 1+ positive	206	4074	4433	5123	1071	1506	17,180
Children <12	7483	23,257	24,045	24,686	25,408	25,050	32,307
Children 12–16	4814	15,503	16,790	18,098	19,012	19,294	22,250
OR + N + S positives	124	4051	2263	695	33	1382	9543
OR + N positives	12	547	2535	4353	1036	244	8842
Patient-facing participants	3335	9464	10,046	10,069	11,103	11,437	15,213

### Mathematical representation of data

Suppose we have a set of n individuals (participants), indexed i,j,…,∈[n] , where we use the notation [k] to stand for the set of integers from 1 to k inclusive. These individuals are members of m households, and we represent the a th household using a set of individual indices $H_{a}$ . These are specified such that each individual is in exactly one household, so formally

$$H_{a}\subseteq [n],\;\forall \;a\in [m],\;H_{a}\cap H_{b}=\emptyset ,\;\forall \;a\in [m],\;b\in [m]\setminus \{a\},\;\overset{m}{\underset{a=1}{⋃}}H_{a}=[n]$$

The size of the a th household is then $n_{a}=|H_{a}|$ . The a th household is visited at a set of times $T_{a}$ , and for each $t\in T_{a}$ we let $x_{i,t}$ be the length- p feature vector (also called covariates) associated with the i th individual at time t , and $y_{i,t}$ be the test result so that $y_{i,t}=1$ if the swab is positive and $y_{i,t}=0$ if not. Note that not all $i\in H_{a}$ will register a valid observation for features and swab results for each $t\in T_{a}$ .

We let a tranche be defined by a time interval $T=[t_{1},t_{2})$ , and the household $H_{a}$ will appear in the analysis associated with the tranche T if $T_{a}\cap T\neq \emptyset$ . For the analysis that we will perform, we require a method for associating a unique positivity and feature vector with each individual for the duration of the tranche. Under our modelling assumptions, the following definition of tranche positivity is most natural. For each household $H_{a}$ associated with tranche T ,

(1) $$\forall i\in H_{a},\;y_{i}=\left\{ \begin{array}{ccc} 1 & \mathrm{if}\;\exists t,y_{i,t}=1\;\; & \;\min\{\tau |\exists j\in H_{a},y_{\;j,\tau }=1\}\in T\\ 0 & \mathrm{otherwise} \end{array}\right.$$

This means that we associate every positive in the household with the tranche in which the first positive appears in that household. Such an approach would need revision for a situation where individuals were infected a large number of times (i.e. common reinfection) or if incidence were so high that a significant number of households would be expected to have multiple introductions, but we do not see these scenarios in our data. For features, the appropriate rule will depend on the feature. An example such rule for the case where there is only one feature $x_{i,t}\in \{0,1\},\forall i,t$ would be

$$x_{i}=\max\{x_{i,t}\;|\;t\in T_{a}\cap T\}$$

i.e. we take this feature to be 1 if it is measured as 1 at any point during the tranche in question.

### Exploratory analysis of density and ages

**Figure 2.**
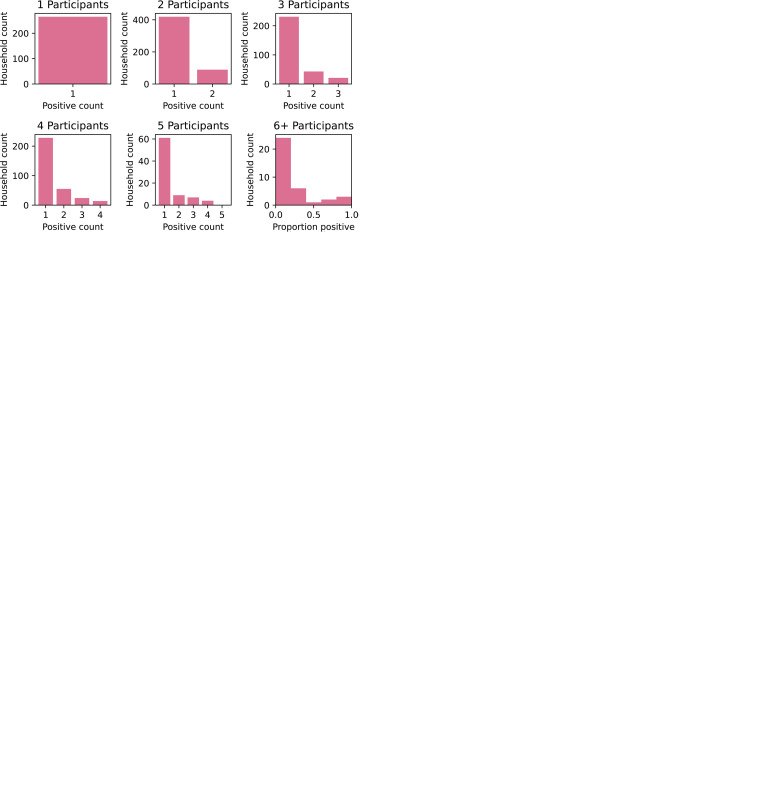
Histograms of household attack rates. (a) Tranche 1, (b) Tranche 2, (c) Tranche 3, (d) Tranche 4, (e) Tranche 5, and (f) Tranche 6.

**Figure 3.**
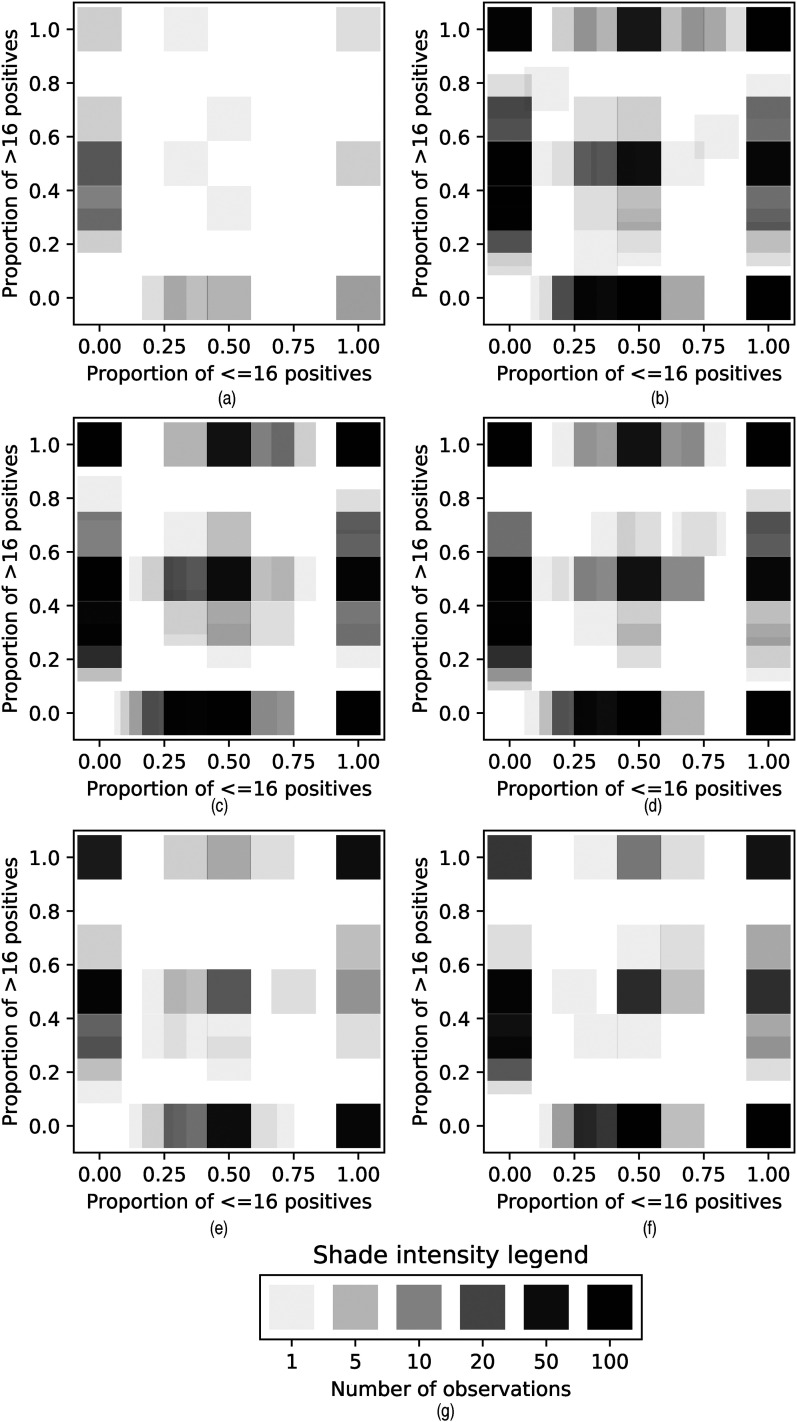
Kernel density plots showing proportion of positives in different age classes in households. (a) Tranche 1, (b) Tranche 2, (c) Tranche 3, (d) Tranche 4, (e) Tranche 5, (f) Tranche 6, and (g) Legend.

An important part of our analysis will be consideration of counts/proportions of households with a given composition of cases displayed as histograms as shown in Figure 2, and density plots as shown in Figure 3.

The heights of the histogram bars are given by

$$Z_{k,\ell }=\sum_{a=1}^{m}1_{\left\{n_{a}=\ell \right\}}1_{\left\{\sum_{i\in H_{a}}y_{i}=k\right\}},\;\ell \in \{2,3,4,5,6\},\;k\in \{0,\ldots ,\ell \}$$

where 1 stands for the indicator function. Verbally, $Z_{k,\ell }$ is the count of households of size ℓ with k participants testing positive.

The density plots are obtained by considering some feature (in this case, age) that takes values 0 or 1 . We then form a point $r_{a}\in [0,1{]}^{2}$ for each household $H_{a}$ such that

$$\sum_{i\in H_{a}}1_{\left\{y_{i}=1\right\}}>0,\;\sum_{i\in H_{a}}1_{\left\{x_{i}=1\right\}}>0,\;\sum_{i\in H_{a}}1_{\left\{x_{i}=0\right\}}>0$$

through the definition

$$r_{a}=\left(\frac{\sum_{i\in H_{a}}1_{\left\{y_{i}=1\;x_{i}=1\right\}}}{\sum_{i\in H_{a}}1_{\left\{x_{i}=1\right\}}},\;\frac{\sum_{i\in H_{a}}1_{\left\{y_{i}=1\;x_{i}=0\right\}}}{\sum_{i\in H_{a}}1_{\left\{x_{i}=0\right\}}}\right)$$

Then we can construct a kernel density estimate in the usual way by summing then normalising kernel functions around the points, in particular the width- w square kernel function

$$K(r,r_{a})=1_{\left\{\|r-r_{a}{\|}_{\infty }<w\right\}}$$

We use age (16 years old and under versus over 16 years old) as the feature in making the density plots in Figure 3.

**Figure 4.**
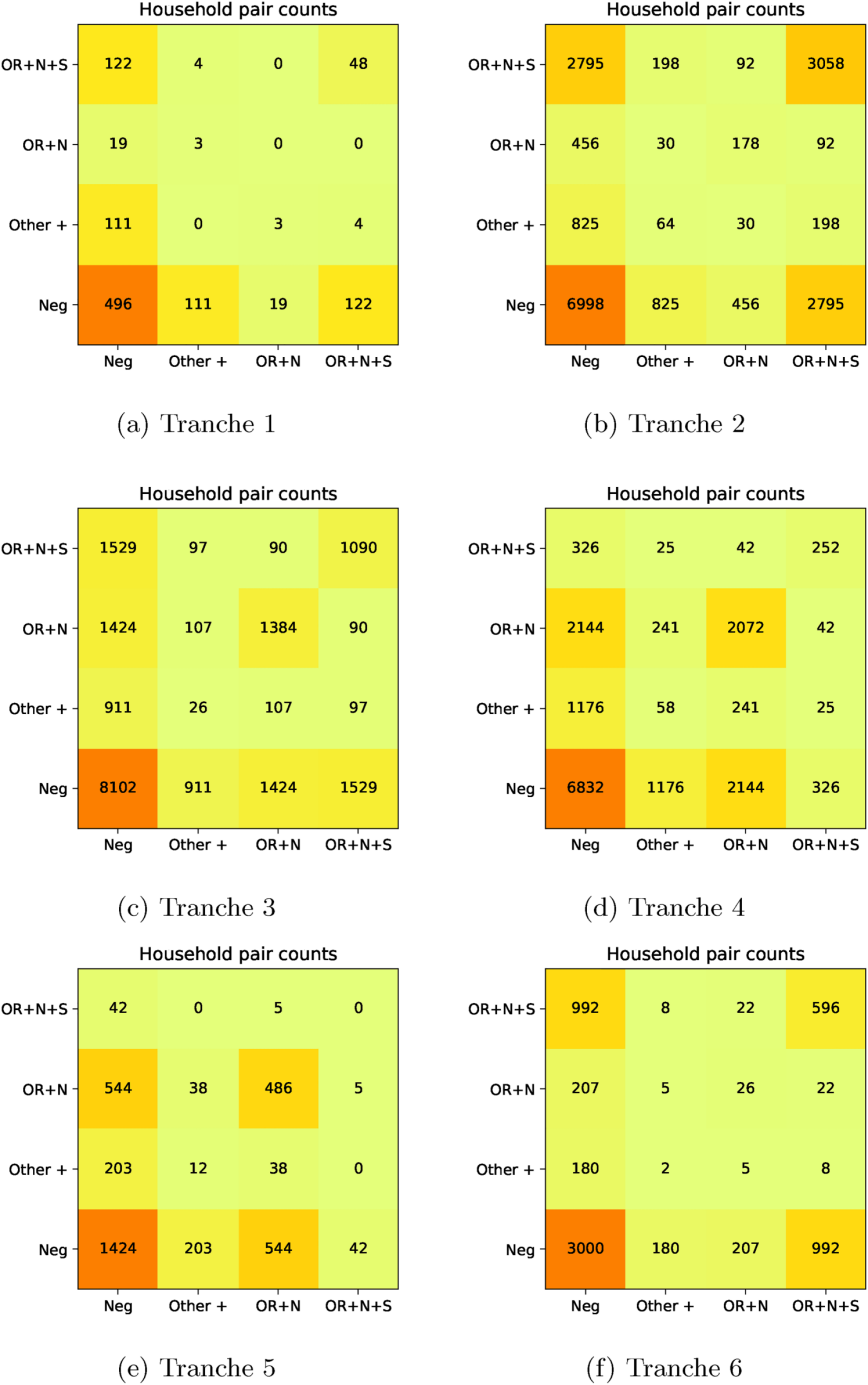
Pair counts for PCR gene positivity patterns. (a) Tranche 1, (b) Tranche 2, (c) Tranche 3, (d) Tranche 4, (e) Tranche 5, and (f) Tranche 6.

### Residual analysis and gene positivity pattern

We are also interested in tabulation of features and positives in households in a manner that allows their clustering to be assessed. In particular, this involves calculation of Pearson residuals for the within-household pairs of features and positives. Let $x_{i}$ be the feature for individual i that takes values with generic labels A,B,… (here mainly patterns of PCR target positivity and negativity indicative of viral strain). We are then interested in the table of pairs of individuals in households in the set H⊆[m] with certain properties,

$$Y_{AB}=\sum_{a\in H,i\in H_{a},j\in H_{a}\setminus \{i\}}1_{\left\{x_{i}=A\right\}}1_{\left\{x_{j}=B\right\}}$$

Verbally, $Y_{AB}$ is the count in the sample of A – B pairs of distinct individuals in households from the set of households under consideration. On its own, this does not indicate whether A and B are more strongly associated with each other in households than would be expected from their overall prevalence in the household population. If we let

$$z_{A}=\sum_{a\in H,i\in H_{a}}1_{\left\{x_{i}=A\right\}}$$

then under the null hypothesis of independence, ${\hat{\pi }}_{A}=z_{A}/|H|$ is the maximum likelihood estimator for the population probability of being in state A and we can then construct an ‘expected’ table corresponding to each household pair having independent state with elements

$$E_{AB}={\hat{\pi }}_{A}{\hat{\pi }}_{B}\sum_{a\in H}n_{a}(n_{a}-1)$$

The Pearson residual associated with the (A,B) th table entry is then

(2) $$R_{AB}=\frac{Y_{AB}-E_{AB}}{\sqrt{E_{AB}}}$$

In simpler contexts, such residuals are typically asymptotically standard normal under the null hypothesis.^37^ For our case, this simple result does not follow straightforwardly, but if we consider a sampled household H , let $X_{i}$ be the random variable state of the i th household member, and let

$$Z_{A}=\sum_{i\in H}1_{\left\{X_{i}=A\right\}}$$

then the moment generating function for the random vector $Z=(Z_{A})$ under the assumption of independence will be the multinomial

$$M_{Z}(t)={\left(\sum_{A}\pi_{A}e^{t_{A}}\right)}^{\left|H\right|}$$

We can then calculate moments of the distribution of pairs through differentiation of this function, for example

$$E\left[Z_{A}\left(Z_{B}-1_{\left\{A=B\right\}}\right)\right]={\frac{\partial^{2}M}{\partial t_{A}\partial t_{B}}|}_{t=0}=\pi_{A}\pi_{B}n(n-1)$$

And so we can see that $R_{AB}$ as in (2) will be 0 where there is no correlation between states at the household level. While explicit calculation of $\mathrm{Var}(Z_{A}(Z_{B}-1_{\left\{A=B\right\}}))$ to determine its asymptotic distribution in the case of many households is beyond the scope of the current work, we believe that this would be an interesting direction for future study. Nevertheless, due to the arguments presented above we can interpret larger values of $R_{AB}$ as indicative of more positive correlation between states at the household level and vice versa.

**Figure 5.**
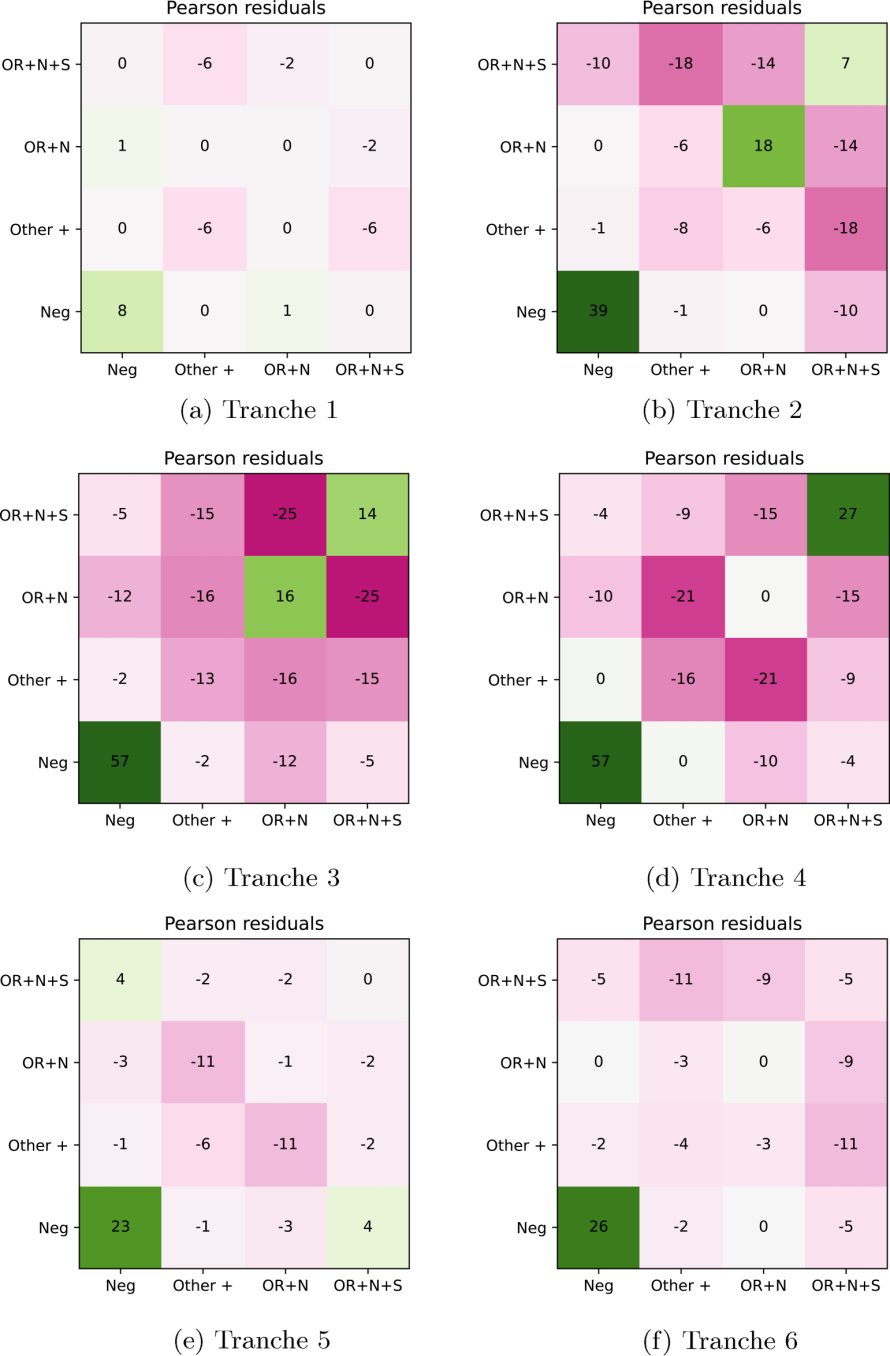
Residual plots for PCR gene positivity patterns. (a) Tranche 1, (b) Tranche 2, (c) Tranche 3, (d) Tranche 4, (e) Tranche 5, and (f) Tranche 6.

Here we will use pattern of PCR target failure as a feature and the restriction of households to those in which there is at least one infection (to avoid domination of the tables by all-negative households), that is

$$H=\left\{a\in [m]|\sum_{i\in H_{a}}y_{i}>0\right\}$$

to produce the plots in Figure 4 and Figure 5.

There are three main patterns of gene positivity that we are interested in: **OR + N + S**, which is generally seen in common pre-Alpha variants and the Delta variant; **OR + N**, which is associated with the Alpha variant; or **Other**, which is usually indicative of too low a viral load to be confident in strain. Where an individual is positive on multiple visits with varying PCR gene positivity patterns, here and throughout we consider the *maximal* pattern, that is, that containing the least target failures. So for example, an individual with an N + S positive at one visit followed by an OR + N + S positive at the next visit and then an N positive at the next visit would be counted as an OR + N + S positive overall.

### Full probability model

While the more exploratory methods above are useful for formulating hypotheses, the main part of our analysis will be household regression, using time, household size and individual features to predict positivity. We start by defining a vector and matrix for each household $H_{a},a\in [m]$ ,

(3) $$y_{a}\;:\;=(y_{i}{)}_{i\in H_{a}},\;X_{a}\;:\;=[(x_{i}{)}_{k}{]}_{i\in H_{a},k\in [p]}$$

Note that the outcomes of swab positivity are not independent of each other due to transmission within households, but otherwise the households are selected as uniformly as possible from the population. This means that an independent-households assumption is appropriate, in which we write the likelihood function as

(4) $$L(\theta )=\prod_{a\in [m]}P_{y_{a}}(X_{a},\theta )$$

Here, θ is a vector of model parameters, and $P_{y}$ is a function mapping a household feature matrix and set of model parameters onto a probability of a given set of household positivity outcomes. We can derive a set of equations for such probabilities from equation (4) of Addy^9^ as by Kinyanjui and House,^38^ and which we present now with some explanation but not a formal derivation of all components.

We will consider the relevant equations for a household H of size n with outcome vector y and feature matrix X (i.e. suppressing the household index a to simplify notation). In particular, given a map $\iota \;:\;\{0,1{\}}^{n}\to \{1,\ldots ,2^{n}\}$ , we will be able to form the vector $P=(P_{\iota (y)}{)}_{y\in \{0,1{\}}^{n}}$ of probabilities of different outcomes in the household. This will be a solution to the set of linear equations

(5) B(θ)P=1

where 1 is a length- $2^{n}$ vector of all ones, and $B=[B_{\iota (\nu ),\iota (\omega )}{]}_{\nu ,\omega \in \{0,1{\}}^{n}}$ , which has

(6) $$B_{\iota (\nu ),\iota (\omega )}=B_{\nu ,\omega }=\frac{1}{\prod_{\;j\in H}\Phi {\left(\sum_{i\in H}(1-\nu_{i})\lambda_{ij}\right)}^{\omega_{j}}Q_{\;j}^{1-\nu_{j}}},\;\nu \leq \omega \in \{0,1{\}}^{n}$$

and other elements equal to zero, where we write ≤ between vectors to stand for the statement that each element on the left-hand side is less than or equal to the corresponding element on the right-hand side. The associated condition imposes that each ν above will correspond to a sub-epidemic of ω meaning that equation (5) can be solved iteratively. There are then three main ingredients of the transmission model that we will enumerate below and in doing so define the terms in equation (6).

The first model component is the probability of avoiding infection from outside; for the i th individual this is

(7) $$Q_{i}=e^{-\Lambda_{i}},\;\Lambda_{i}=\Lambda e^{\alpha \cdot x_{i}}=e^{\alpha_{0}+\alpha \cdot x_{i}}$$

In the language of infectious disease modelling, $\Lambda_{i}$ is the cumulative force of infection experienced by the i th individual. Then $\exp\;(\alpha_{k})$ is the relative external exposure associated with the k th feature/covariate, meaning that it is the multiplier in front of the baseline force of infection, which is that for an individual whose feature vector is all zeros, 0 . This baseline probability of *avoiding* infection from outside is then

(8) $$q=\exp\;(-\Lambda )=\exp\;(-\exp\;(\alpha_{0}))$$

Because this is often much closer to 1 than to 0 , we will report the probability of *being* infected from outside the household as a percentage, that is, (1−q)×100% will be given in the figures and tables. We will present this alongside the relative external exposures that are elements of the vector α , although it would also be possible to use (8) to relate q to the baseline force of infection Λ or intercept of the linear predictor, $\alpha_{0}$ . Note that some care must be taken in interpretation of this variable when the data are split into time periods as in this work, since to appear as a household with at least one positive in one tranche, it is necessary to appear as a household with no positives in the previous tranches for which the household was in the study. Values of 1−q will typically be low enough here that this conditional dependence is not strong, but this might not be true at higher levels of incidence for the same design.

The second component of the model is variability in the infectiousness at the individual level, usually interpreted as arising from the distribution of infectious periods. Suppose, in particular, that a household has just one susceptible and one infectious individual, and that the infectious individual exerts a force of infection λ on the susceptible for a random period of time T . Let the cumulative force of infection be

(9) $$C(t)=\int_{u=0}^{\min(T,t)}\;\lambda \;du$$

The first step in analysing this model is to apply the Sellke^39^ construction, where the susceptible individual picks a random variable Ξ∼Exp(1) and infection happens once C(t)>Ξ , or no infection happens if C(T)<Ξ . To see why this is equivalent to infection at a rate λ , take (9) and note that

$$\mathrm{Pr}(\Xi >C(t+\delta t)|\Xi >C(t))=\frac{\int_{0}^{C(t+\delta t)}\exp(-\xi )\;\int \xi }{\int_{0}^{C(t)}\exp(-\xi )\;d\int \xi }=1-\lambda \delta t+o(\delta t)$$

The furthest right expression in this equation is what we mean by infection at a rate.

Using $F_{X}$ to stand for a cumulative distribution function and $f_{X}$ for a probability density function of a random variable X , we have the total probability of avoiding infection as

(10) $$\mathrm{Pr}(\Xi >C(T))=\int_{0}^{\infty }F_{\Xi }(\lambda t)f_{T}(t)\;\int t=\int_{0}^{\infty }e^{-\lambda t}f_{T}(t)\;\int t=L[f_{T}](\lambda )=\;:\;\Phi (\lambda )$$

where L stands for Laplace transformation. We can then use this result to write down the probabilities of different outcomes in a two-person household without covariates:

$$\mathrm{Pr}(y=(0,0))=Q^{2},\;\mathrm{Pr}(y=(0,1))=\mathrm{Pr}(y=(1,0))=Q(1-Q)\Phi (\lambda )$$

which are expressions that can also be obtained from (6). A more general argument is presented by Ball^8^ for the full system of equations, but the expressions above should give some intuition for why these hold.

For our modelling, we assume that each individual picks an infectious period from a unit-mean Gamma distribution since the equations are not sensitive to the mean and this therefore provides a natural one-parameter distribution with appropriate support. The Laplace transform of this as used in (6) is

(11) $$\Phi (s)=(1+\vartheta s{)}^{-1/\vartheta }$$

The parameter ϑ is the variance of the Gamma distribution, that is, it is larger for more individual variability.

The third component of the model is the infection rate from individual j to individual i ,

(12) $$\lambda_{ij}=n^{\eta }\lambda \sigma_{i}\tau_{j}=n^{\eta }\lambda e^{\beta \cdot x_{i}}e^{\gamma \cdot x_{j}}=e^{\beta \cdot x_{i}}e^{\gamma_{0}+\eta \log\;(n)+\gamma \cdot x_{j}}$$

In this equation, λ is the baseline rate of infection; $\sigma_{i}=e^{\beta \cdot x_{i}}$ is the relative susceptibility of the i th participant, and $\exp\;(\beta_{k})$ is the relative susceptibility associated with the k th feature; $\tau_{j}=e^{\gamma \cdot x_{j}}$ is the relative transmissibility of the j th participant, and $\exp\;(\gamma_{k})$ is the relative transmissibility associated with the k th feature/covariate. As can be seen from (12), we can interpret log(λ) as the intercept of the linear predictor for transmissibility. The term $n^{\eta }$ is a modelling approach to the effect of household size usually attributed to Cauchemez et al.^10^; as can be seen from (12), this is equivalent to taking log(n) as a covariate for transmissibility. Experience with fitting these models^40^ suggests that it is a good idea to impose hard bounds on the Cauchemez parameter, that is, insist that $\eta \in [\eta_{\min},\eta_{\max}]$ , meaning that here we will treat η separately from other parameters.

### Model variables and fitting

We now enumerate all of the model parameters, distinguishing between the ‘natural’ representations of parameters that sit in R and transforms of natural parameter space $R^{\kappa }$ that are most epidemiologically interpretable and therefore suitable for reporting. Since Λ , λ and ϑ have positive support, we can use logarithmic and exponential functions to transform between epidemiological and natural parameters. As noted above, we want η to have compact support, and so note that the function tan:[−π/2,π/2]→R and its inverse can be used. We choose $\eta_{\min}=-2$ and $\eta_{\max}=2$ , meaning that our natural parameter vector is

$$\theta =(\log\;(\Lambda ),\log\;(\lambda ),\log\;(\vartheta ),\tan\;(\pi \eta /4),\alpha ,\beta ,\gamma )\in R^{\kappa }$$

The first part of this parameter vector is the external force of infection, with natural representation $\alpha_{0}=\log\;(\Lambda )$ . Here we will quote the baseline probability of infection from outside as a percentage, which is (1−q)×100% for q as in (8).

The second part of the parameter space relates to baseline within-household transmission with natural representation $\gamma_{0}=\log\;(\lambda )$ , log(ϑ) , and tan(πη/4) , where we use this transform for η to make a hard constraint of epidemiologically meaningful values. For interpretability, we work with probabilities of infection by household size, which from generalising (10) to a size-scaled transmission rate are

(13) $$p_{n}=1-\Phi (n^{\eta }\lambda )$$

Such quantities have been called Susceptible-Infectious Transmission Probabilities (SITP) by, for example, Fraser et al.,^5^ who estimated values close to 20% from historical data on the 1918 influenza pandemic.

The third part are features, where we consider:

- Three age groups: 2–11 years old; 12–16 years old; and older.
- Working in a patient-facing role or not.
- Pattern of PCR gene target positivity: OR + N + S, which is associated with pre-Alpha variants and the Delta variant; OR + N, which is associated with the Alpha variant; or other, which is usually indicative of too low a viral load to be confident in strain.

We assume that age and working in a patient-facing role have an association with external risk, leading to natural parameters $\alpha_{2-11}$ , $\alpha_{12-16}$ and $\alpha_{\mathrm{PF}}$ ; that age has an association with susceptibility, leading to natural parameters $\beta_{2-11}$ and $\beta_{12-16}$ ; and that age and gene positivity in PCR have an association with transmissibility, leading to natural parameters $\gamma_{2-11}$ , $\gamma_{12-16}$ , $\gamma_{\mathrm{OR}+N}$ and $\gamma_{\mathrm{oth}}$ . For any natural parameter r , we will report the multiplicative effect exp(r) .

Model fitting was performed in an approximate Bayesian framework using the Laplace approximation. As noted above, households of size 7 and larger were excluded from the analysis partly because these are often very different in composition from smallerhouseholds, and partly because of the numerical cost of solving a linear system of size $2^{n}$ We combine the likelihood (4) with a standard normal prior on natural parameters, $\theta ∼N_{\kappa }(0,I)$ . Sensitivity of results to this prior was considered for different variances and revealed essentially no impact on the highly identifiable parameters such as Λ and λ , and that while a higher variance could slightly reduce the shrinkage of effect sizes towards zero, it could also lead to instability in fitting, meaning that this prior achieves regularisation of the inference problem without excessive bias. The maximum a posteriori estimate was obtained using multiple restarts of a Quasi-Newton optimiser. The Hessian was calculated numerically for the natural parameters and used in the Laplace approximation to the posterior on the natural parameters. The credible intervals (CIs) are then transformed from natural to epidemiologically interpretable parameters.

### Data processing and software implementation

The analysis was carried out on the ONS Secure Research Server in the Python 3 language. To illustrate issues with data processing, note that the ‘flat’ form for the data extracted from the database after cleaning takes a form like:

**Table:** 

HID	PID	Visit Date	Age	Test Result	Work PF	Pattern
...						
123	456	2020-10-02	8	Negative	No	NA
123	457	2020-10-02	38	Negative	No	NA
123	456	2020-10-10	8	Negative	No	NA
123	457	2020-10-10	38	Positive	No	OR+N+S
123	456	2020-10-17	9	Positive	No	OR+N+S
123	457	2020-10-17	38	Negative	No	NA
...						
124	458	2021-02-15	53	Negative	Yes	NA
...						

In particular, there is a hierarchical structure to the data. Households, each with a unique household ID in the HID column, have a number of study participants with a unique participant ID in the PID column, and each participant being visited on a number of dates as in the Visit Date column. Each visit will have associated participant features (e.g. as in the Age column above) and a Test Result.

The large size of this flat file (slightly under three million rows) means that it is advantageous to use specialist libraries, in this case *pandas*^41,42^ together with *NumPy*.^43^ To deal with the nested structure of the data, we used the ‘split-apply-combine’ paradigm that this library encourages by analogy with SQL operations. In the example above, this would involve first associating each participant with an age using pandas.groupby(′PID′) and pandas.DataFrame.apply(numpy.min), and then producing an array of ages for each household using pandas.groupby(′HID′) and pandas.DataFrame.apply(numpy.array). A similar approach is possible for test results and multiple features.

Apart from data processing, the main computational cost of the analysis is the linear algebra associated with solving (5), particularly for larger households. Due to portability, this was carried out in NumPy on the ONS system, however we found that implementation in *Numba*^44^ can generate significant speed-ups, as might use of GPU hardware through use of, for example, *PyTorch*.^45^

Access to ONS CIS data is possible via the Office for National Statistics’ Secure Research Service, and Python code demonstrating the methodology applied to publicly available data is at https://github.com/thomasallanhouse/covid19-housefs.

## Results and discussion

### Exploratory analysis

Figure 2 shows the distribution of positives in households; comparison with Table 2 shows that the number of households with two or more positives are much greater than would be expected under the assumption of independence. In fact, some histograms even take a bimodal ‘U’-shape.

This multi-modality is even more apparent in the kernel plots in Figure 3, which also demonstrate that it is common to see households with only child positives, only adult positives, or both. In particular, this suggests that both children and adults can be responsible for bringing infection into the household. While some of the child-infection-only households could arise due to failure of ascertainment of an adult infection in the household, this is unlikely to be true for most, meaning, the introduction of infection to the household would have been due to a child (and vice versa for adult-infection-only households).

### Residual analysis

The pair counts and Pearson residual analysis – applied to the maximal PCR target gene positivity pattern being OR + N + S, OR + N, other positive, or negative – are shown in Figures 4 and 5. The pair counts show at the household level the replacement of the OR + N + S pattern as the main source of positive pairs in households with the OR + N pattern, and then the return of the OR + N + S pattern. We also see from the residual plots that while there is positive correlation of (OR + N + S) − (OR + N + S) and (OR + N) − (OR + N) pairs, as well as of negative-negative pairs, there is a negative correlation associated with (OR + N + S) − (OR + N) pairs and also between pairs involving any other positive pattern. While this analysis is not mechanistic or causal, we expect that the main factor generating correlation/clustering of positives in households is transmission. As such, the results are consistent with our understanding of the sweeps of the Alpha and Delta variants as arising due to these being more transmissible strains than those that they replaced.

### Regression analysis

**Figure 6.**
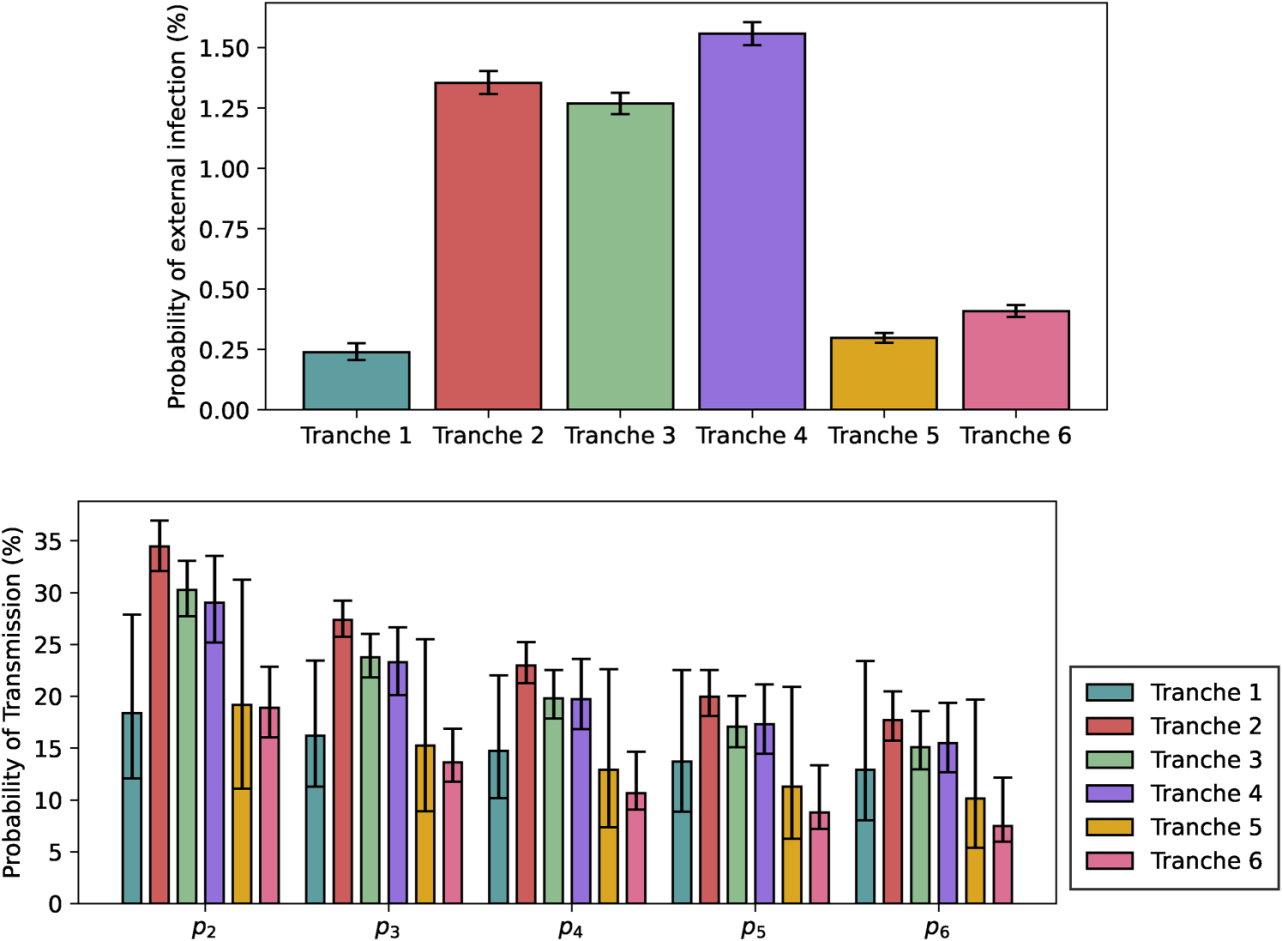
Visualisation of the fitted model. Top: Baseline probability of infection from outside. Bottom: Per-pair baseline probabilities of secondary transmission within the household, not including tertiary transmission effects.

**Figure 7.**
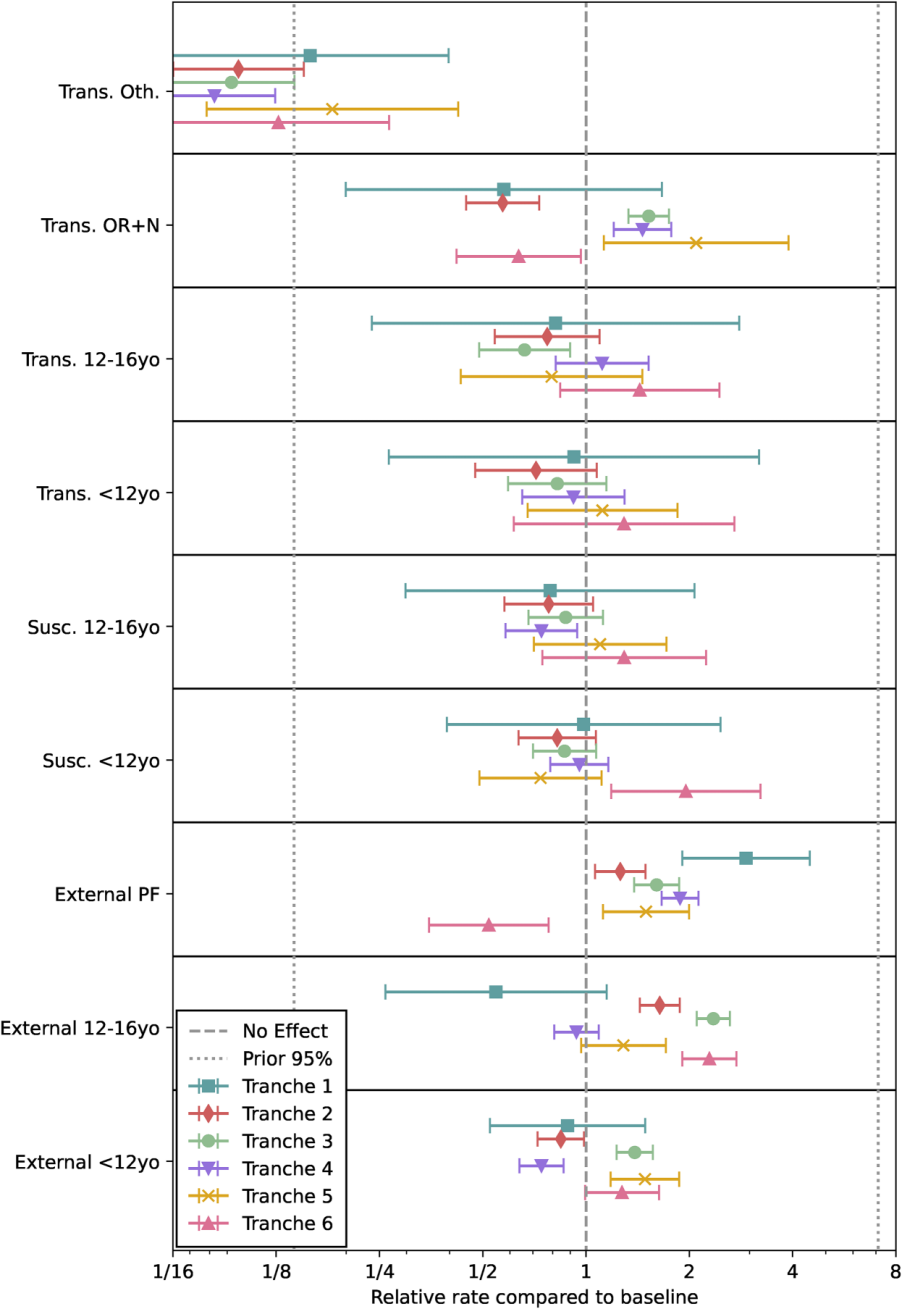
Visualisation of the fitted model. Relative effects on transmission, susceptibility and external exposure compared to baseline of an adult not working in a patient-facing role with OR + N + S maximal PCR gene positivity pattern if positive. ‘Trans.’ stands for relative transmissibility, ‘Susc.’ for relative susceptibility and ‘External’ for relative external exposure.

The regression analysis has its outputs shown in Table 3, Figures 6 and 7. We now present these in order.

**Table 3.** The parameter point estimates and CIs.

	Tranche 1	Tranche 2	Tranche 3	Tranche 4	Tranche 5	Tranche 6
1−q	0.237 (0.205, 0.274) %	1.35 (1.31, 1.4) %	1.27 (1.22, 1.31) %	1.56 (1.51, 1.61) %	0.296 (0.277, 0.317) %	0.408 (0.384, 0.433) %
$p_{2}$	18.4 (12.1, 27.9) %	34.5 (32.1, 37.0) %	30.2 (27.7, 33.1) %	29.0 (25.2, 33.5) %	19.2 (11.1, 31.3) %	18.9 (16.0, 22.9) %
$p_{3}$	16.2 (11.3, 23.4) %	27.4 (25.7, 29.2) %	23.8 (21.8, 26.0) %	23.3 (20.1, 26.7) %	15.3 (8.92, 25.5) %	13.6 (11.8, 16.9) %
$p_{4}$	14.8 (10.2, 22.0) %	23.0 (21.3, 25.2) %	19.8 (17.9, 22.5) %	19.7 (16.8, 23.6) %	12.9 (7.38, 22.6) %	10.7 (9.09, 14.6) %
$p_{5}$	13.7 (8.86, 22.5) %	20.0 (18.1, 22.5) %	17.1 (15.1, 20.0) %	17.3 (14.5, 21.1) %	11.3 (6.25, 20.9) %	8.79 (7.22, 13.3) %
$p_{6}$	12.9 (8.06, 23.4) %	17.7 (15.7, 20.5) %	15.1 (13.0, 18.6) %	15.5 (12.7, 19.4) %	10.1 (5.4, 19.7) %	7.48 (5.97, 12.2) %
$\exp\;(\alpha_{2-11})$	0.883 (0.525, 1.49)	0.845 (0.723, 0.987)	1.39 (1.23, 1.56)	0.742 (0.64, 0.86)	1.48 (1.18, 1.87)	1.27 (0.993, 1.63)
$\exp\;(\alpha_{12-16})$	0.546 (0.26, 1.15)	1.64 (1.44, 1.87)	2.35 (2.1, 2.63)	0.938 (0.807, 1.09)	1.29 (0.967, 1.71)	2.29 (1.91, 2.74)
$\exp\;(\alpha_{\mathrm{PF}})$	2.93 (1.91, 4.49)	1.26 (1.06, 1.49)	1.61 (1.38, 1.87)	1.88 (1.66, 2.13)	1.5 (1.12, 2.0)	0.521 (0.349, 0.778)
$\exp\;(\beta_{2-11})$	0.984 (0.393, 2.46)	0.824 (0.636, 1.07)	0.865 (0.7, 1.07)	0.956 (0.787, 1.16)	0.737 (0.49, 1.11)	1.95 (1.18, 3.22)
$\exp\;(\beta_{12-16})$	0.786 (0.298, 2.07)	0.778 (0.578, 1.05)	0.872 (0.68, 1.12)	0.741 (0.583, 0.943)	1.1 (0.704, 1.71)	1.29 (0.746, 2.24)
$\exp\;(\gamma_{2-11})$	0.922 (0.266, 3.2)	0.715 (0.476, 1.07)	0.824 (0.593, 1.15)	0.919 (0.652, 1.29)	1.12 (0.676, 1.85)	1.29 (0.615, 2.71)
$\exp\;(\gamma_{12-16})$	0.815 (0.237, 2.8)	0.771 (0.542, 1.1)	0.662 (0.488, 0.899)	1.11 (0.815, 1.52)	0.794 (0.432, 1.46)	1.43 (0.841, 2.45)
$\exp\;(\gamma_{\mathrm{OR}+N})$	0.576 (0.199, 1.67)	0.572 (0.447, 0.731)	1.52 (1.33, 1.75)	1.46 (1.2, 1.77)	2.09 (1.13, 3.89)	0.636 (0.419, 0.965)
$\exp\;(\gamma_{\mathrm{CT}-\mathrm{oth}})$	0.157 (0.062, 0.398)	0.097 (0.0626, 0.15)	0.0926 (0.0607, 0.141)	0.0826 (0.055, 0.124)	0.182 (0.0783, 0.424)	0.127 (0.0604, 0.267)

The baseline external probabilities of infection shown in the top plot of Figure 6 follow the rough pattern that would be expected from community prevalence and Tranche duration, with the notable exception of Tranche 6, when it is likely that vaccination significantly reduced the infection risk despite high prevalence. In terms of the baseline probabilities of within-household transmission in the bottom plot of Figure 6, these are largely consistent in terms of overlapping credible intervals for Tranches 2 to 4, with Tranche 6 noticeably lower and with credible intervals that do not overlap with those for Tranches 2 to 4, likely due to the impact of vaccination (and despite the emergence of the Delta variant). The low-prevalence Tranches 1 and 5 have large credible intervals, so are hard to distinguish statistically from the other tranches, despite having lower point estimates. It is worth noting that for periods of low prevalence following periods of high prevalence, we expect lower viral loads on average as noted by Hay et al.,^46^ and this might impact on overall transmissibility estimates.

Turning to Figure 7, we see that ‘other’ patterns of gene positivity (besides OR + N and OR + N + S) are consistently associated with much lower transmissibility, as would be expected given target failure is more likely at lower viral loads.^47^ We also see lower transmissibility of OR + N prior to the emergence of the Alpha variant, since S-gene target failure would have been associated with lower viral loads at that point as well, but higher transmissibility for this pattern after the emergence of Alpha but before the emergence of Delta. After the emergence of Delta, the OR + N pattern is associated with lower transmissibility than OR + N + S, as would be expected.

In terms of child susceptibility and transmissibility, there is no strong evidence for an effect. While it is plausible that non-vaccination of children would lead to increasing their relative susceptibility at later times, this is consistent with the Tranche 6 results but not strongly evidenced by them.

For patient-facing staff, external risk of infection has been consistently high until reduced in Tranche 6, most likely due to the impact of vaccination. For children, external risk of infection is generally raised compared to baseline when schools are open, with the exception of primary school aged children before the emergence of Alpha. Whether this change in association is due to some causal factor not accounted for here, or is related to the new variants spreading more efficiently amongst young children than wildtype, requires further investigation.

### Limitations and directions for future work

While we have taken many steps to ensure that the results presented here are as robust as possible, there are key limitations to the analysis that need to be borne in mind. The main one of these is failures in ascertainment of positives and other missingness in the longitudinal design in question. The most likely consequence of this will be to depress susceptible-infectious transmission probability estimates. One theoretical approach to deal with this would be imputation of the transmission tree as suggested by Demiris and O’Neill,^11^ but this is likely to be too computationally intensive to be practical in the current context. Another would be analytical work to include failure of ascertainment into the likelihood function as in House et al.,^15^ however it is unclear how to model ascertainment probabilistically in a tractable manner. A data-driven approach would be to try to include positives from other sources such as Test and Trace case data or self-reported episodes of illness. There is also a harder to quantify potential bias of non-participation in the study, particularly if this is with respect to some factor that is not measured.

Another important limitation is the possibility that other features, for example the geographical region that households are in, more detailed information about viral load and symptoms, or information about the physical structure of the household, might play an important explanatory role in the associations observed. Finally, there are possible refinements of the work: trends in external infection over time could be modelled as a flexible functional form (e.g. a spline as in^24^); extra features could be added, and features selected using formal criteria, including relaxing of the Cauchemez assumption to allow transmission probabilities to depend in a general manner on household size, and explicit correction to attack rates due to shrinking and growing epidemics could be made as proposed by Ball and Shaw^48^ and Shaw^49^; model parameters – for example, baseline transmission probabilities – could be shared across tranches; the work could be extended to Wales, Scotland and Northern Ireland; more formal analysis of causal pathways could be performed; and improvements could be made in implementation data processing, model evaluation through improved linear algebra, and fitting algorithm. These and other directions should be the subject of future studies.

## Notes

1. https://www.ndm.ox.ac.uk/covid-19/covid-19-infection-survey/protocol-and-information-sheets; ISRCTN number ISRCTN21086382; The study received ethical approval from the South Central Berkshire B Research Ethics Committee (20/SC/0195).

2. https://www.ndm.ox.ac.uk/covid-19/covid-19-infection-survey/case-record-forms
